# Editorial: Accessing Conceptual Representations for Speaking

**DOI:** 10.3389/fpsyg.2016.01216

**Published:** 2016-08-18

**Authors:** Ian FitzPatrick, Peter Indefrey

**Affiliations:** ^1^Institut für Sprache und Information, Heinrich Heine University DüsseldorfDüsseldorf, Germany; ^2^Centre for Cognitive Neuroimaging, Donders Institute for Brain, Cognition, and Behaviour, Radboud University NijmegenNijmegen, Netherlands

**Keywords:** language production, conceptual representation, semantics, lexical access, conceptual attributes

Systematic investigations into the role of semantics in the speech production process have remained elusive. This special issue aims at moving forward toward a more detailed account of how precisely conceptual information is used to access the lexicon in speaking and what corresponding format of conceptual representations needs to be assumed. The studies presented in this volume investigated effects of conceptual processing on different processing stages of language production, including sentence formulation, lemma selection, and word form access.

## Conceptual processing for sentence formulation

Using an eye-tracking paradigm in which participants are prompted to describe pictures of two-character transitive events, Ganushchak et al. show that contextually new referents are fixated with priority over contextually old (i.e., given) referents. The time course of the contextual effects on gaze patterns suggests that contextual information might well be taken into account during sentence formulation. Hsiao et al. present data from a sentence production task and a corpus study that show that speakers of Mandarin Chinese are more prone to omitting subject pronouns in their utterances when the subject and object of the sentence are conceptually similar (e.g., both animate or both inanimate) than when they are conceptually dissimilar.

## Relationships between conceptual and lexical activation in monolingual and bilingual speakers

The majority of studies aimed at gaining further insights into classic distractor effects. Harvey and Schnur investigated semantic interference in picture naming and word–picture matching. Using a blocked-cyclical paradigm they show that semantic interference in naming generalizes to novel objects, but semantic interference in word–picture matching does not. This is taken as evidence that semantic interference effects in naming and word–picture matching arise at different processing stages. Naming novel items that corresponded to semantic categories that had been previously encountered in word–picture matching induced semantic interference. The latter result suggests a common origin of semantic interference across tasks.

Bölte et al. investigated the origin of semantic interference effects in the picture–picture paradigm. Participants named pictures of German compound words which were accompanied by categorically or associatively related distractor objects. Categorically related distractors facilitated naming at SOAs at which semantic processing is expected (in this case +200). The authors argue that the absence of semantic interference means that such distractors activate their conceptual-semantic information but do not activate the corresponding lemma.

Vieth et al. investigated semantic interference from distinctive features. Their first experiment showed no evidence that distractors that differed from target items on a distinctive feature (e.g., for HORSE-/zebra/the feature stripes) were processed differently from semantically matched distractors with no distinctive feature differences (e.g., HORSE-/donkey/). Further experiments showed that distractors denoting visible parts of target objects that are also found in other objects (e.g., GOAT—*tail*) slowed down naming of target items. The authors argue that this reflects competition from semantically related items (e.g., other animals with tails).

Damian and Spalek used a picture–word-interference paradigm with distractors that were either unrelated, categorically related, associatively related, or both categorically and associatively related. In addition the authors manipulated the visibility of distractors by presenting them in between forward and backward masks. Results replicate earlier (Finkbeiner and Caramazza, 2006; Dhooge and Hartsuiker, 2010) reports of semantic facilitation (rather than inhibition) for masked distractors. Importantly, however, the picture–word-interference effect did not seem to depend on individual subject differences in the ability to recognize the masked distractors. The authors take these results as more in line with competition threshold accounts (e.g., Piai et al., 2012) for picture–word interference rather than response exclusion accounts (Finkbeiner and Caramazza, 2006; Dhooge and Hartsuiker, 2010).

Hutson and Damian tested a prediction of the response exclusion account of the picture–word-interference effect, namely that for semantically closely related items, priming counteracts buffer-based interference. They found no evidence of degree of semantic relatedness in picture–word-interference. This result, they argue, is difficult to reconcile with either response exclusion accounts (which would need to abandon the notion of conceptual priming from semantically related distractors) or competitive accounts (which would need to postulate opposing effects of conceptual priming and semantic interference canceling each other out).

Two studies investigated relationships between conceptual and word form activation in bilingual speakers. Von Holzen and Mani show that bilinguals implicitly generate labels for pictures simultaneously in their first and second languages. Targets preceded by phonologically related pictures showed lower N400 effects irrespective of whether the phonological relationship was within or between languages. This implies that the non-selected (non-target language) lemma can send activation cascading forward to the phonological level. Correia et al. studied the reverse flow of activation. Using multivariate pattern analysis of EEG data, they show that in bilingual listeners language invariant semantic representations can be decoded around 550 ms following the onset of a spoken word.

## Activation of conceptual attributes

Finally, two studies investigated the role of attribute retrieval in naming. Mulatti et al. show that white noise interferes with naming pictures of objects with typical sounds but not with objects without typical sounds. This suggests that an object's sound attribute is used during lemma retrieval. Lloyd-Jones and Nakabayashi examined the retrieval of object color information using a picture naming and semantic matching task. Their results suggest differential retrieval of color information for object names and object shapes.

## Conclusion

It becomes clear in this volume that effects of conceptual processing extend beyond the conceptual level and can affect many levels of processing. The range of conceptual relationships that are explored is just beginning to be expanded beyond categorical and associative relationships.

## Author contributions

All authors listed, have made substantial, direct and intellectual contribution to the work, and approved it for publication.

## Funding

This research was funded by the Deutsche Forschungsgemeinschaft (DFG) Collaborative Research Centre (CRC) 991.

### Conflict of interest statement

The authors declare that the research was conducted in the absence of any commercial or financial relationships that could be construed as a potential conflict of interest.
